# The Perioperative Use of Dexmedetomidine in Paediatric Patients

**DOI:** 10.3390/children12060690

**Published:** 2025-05-28

**Authors:** Esaias Janse van Rensburg, Laura Indiveri, Palesa Mogane

**Affiliations:** 1 Registrar Anaesthesiology, University of Witwatersrand, Johannesburg 2193, South Africa; 2 Department of Anaesthesiology, Charlotte Maxeke Johannesburg Academic Hospital, Johannesburg 2193, South Africa; laura.indiveri@wits.ac.za; 3 Department of Anaesthesiology, Chris Hani Baragwanath Academic Hospital, Johannesburg 1864, South Africa; palesa.mogane@wits.ac.za

**Keywords:** dexmedetomidine, paediatric anaesthesia, sedation, analgesia

## Abstract

**Background/Objectives**: Dexmedetomidine, an alpha-2 adrenergic agonist, has gained significant attention for its sedative, analgesic, and anxiolytic properties in paediatric anaesthesia. This review explores its pharmacokinetics and pharmacodynamics, perioperative applications and efficacy, and safety profile in paediatric patients. **Findings**: Dexmedetomidine has emerged as a highly effective adjunct in paediatric anaesthesia, offering significant advantages across various perioperative settings. It reduces the need for other anaesthetics and opioids, leading to smoother recoveries with lower postoperative pain and agitation. Studies highlight its role in enhancing procedural sedation, improving patient cooperation, and providing superior analgesia in neuraxial and general anaesthesia. Its neuroprotective properties and stable haemodynamic profile make it particularly valuable in the perioperative and critical care settings. **Conclusions**: Dexmedetomidine has shown a favourable safety and efficacy profile in paediatric anaesthesia when doses are carefully titrated within the ranges recommended in the literature. While its use remains off-label in paediatric populations, increasing clinical experience and evidence support its integration into perioperative protocols.

## 1. Introduction

The use of dexmedetomidine, an alpha(α)-2 adrenergic agonist, as an adjunct to paediatric anaesthesia has amassed great popularity in recent years [1,2,3,4,5]. It is known for its sedative, anxiolytic, and co-analgesic effects, making it an appealing option for paediatric patients [6,7]. In 1999, dexmedetomidine received index approval for sedation use in the adult intensive care unit by the Food and Drug Administration in the United States [8]. The earliest documented use of dexmedetomidine in children stems from a 2002 composite series of case reports [9]. Since its inception, varied indications, dosages, and routes of administration have been employed in a variety of patient groups. An international survey of 791 paediatric anaesthesiologists found that 70% use dexmedetomidine, mainly for procedural sedation, with wide dose variations. Nearly half reported no adverse effects. Among those not using it, most lacked training but were open to adoption [1].

Paediatrics is an understudied population, seldom subject to rigorous clinical trials. As a result, the use of many pharmaceuticals in children is off label. Off label use does not constitute illegal, improper or contraindicated use. Practitioners rely on available evidence, clinical experience, and resources to guide off-label prescribing decisions for the patient’s benefit [10,11]. The use of dexmedetomidine in children is off label [1]. This review explores the reasoning behind dexmedetomidine usage, various dosing strategies, and reviews the existing evidence supporting its safety and effectiveness in paediatric care.

## 2. Pharmacokinetics and Pharmacodynamics

Dexmedetomidine shows a α2: α1-adrenoceptor affinity ratio of 1600:1, eight times more than Clonidine [12]. It may be administered via intramuscular (IM), intravenous (IV), nasal (IN), subcutaneous, buccal, and rectal routes. The bioavailability of oral, buccal and intramuscular administration was found to be 16%, 82% and 104% respectively. For this reason the oral route is not recommended and is due to high first-pass metabolism [13]. In most settings, the IV route is recommended and used [14]. It is highly lipophilic with a high volume of distribution (V_D_) in adults and children. Dexmedetomidine is mainly bound to plasma proteins albumin and α-1 glycoprotein. It crosses the blood brain barrier with ease, with children older than 1 year having a V_D_ similar to that of adults. Metabolism occurs via cytochrome P450 (CYP2A6) and uridine 5′-diphospho-glucuronosyl-transferase liver enzymes (UGT1A4 and UGT2B10) and excretion via bilious and renal routes. With each pass through the liver, 70% of the drug is removed from the circulation. A small amount (less than 1%) is excreted unaltered by the kidneys [15].

Dexmedetomidine is speedily distributed and has an elimination half-life of approximately 120 min. Clearance changes greatly with age. Children beyond infancy have clearance rates closely related to those described in adults (42.1 L.h^−1^.70 kg^−1^ vs. 44.8–52.5 L.h^−1^.70 kg^−1^). Clearance in neonates and infants is decreased (42.4% of adult rates, reaching 84.4% by 1 year old) due to immature elimination pathways [16,17].

By analysing 354 individuals post-cardiac surgery aged 0–22 years (16 months median age), researchers developed a two-compartment population pharmacokinetic (popPK) model. They found that body weight and postmenstrual age significantly influenced drug clearance, with clearance maturing according to a sigmoid Hill function. Importantly, pharmacogenetic factors (UGT1A4, UGT2B10, and CYP2A6 variants) did not significantly affect clearance, suggesting that age and body size are the primary drivers of inter-individual variability in dexmedetomidine pharmacokinetics in children [18]. Conversely, in a younger cohort of 2–72 month old children Guan et al. found that carriers of the T allele at the CYP2A6 rs835309 locus exhibited a 13% lower mean concentration, suggesting enhanced metabolic clearance [19].

Dexmedetomidine elimination half-life decreases with age in children. In preterm neonates (28 to <36 weeks GA), the median half-life is 7.6 h (range: 3–9.1 h), while in term neonates (36 to ≤44 weeks GA), it’s 3.2 h (range: 1–9.4 h) [20]. In infants and children under 2 years, the median is 2.3 h (range: 1.5–3.3 h), and in those aged 2 to 11 years, it drops to 1.6 h (range: 1.2–2.3 h) [21].

An investigation into the pharmacokinetics (PK) of dexmedetomidine in neonates and infants undergoing corrective cardiac surgery involving cardiopulmonary bypass (CPB) found that dexmedetomidine clearance significantly decreases during CPB—by approximately 95% compared to pre-CPB values—and by 50% in the immediate postoperative period. This profound reduction is likely due to diminished hepatic blood flow and hypothermia during CPB. A validated population PK model (NONMEM), using both allometric and linear scaling, was developed and enabled simulation-based dosing strategies to maintain desired steady-state plasma concentrations. Throughout the perioperative period, 96.1% of measured concentrations in validation subjects fell within the model’s predicted 5th–95th percentile intervals. The study highlights the importance of adjusting dexmedetomidine dosing during and after CPB, particularly due to age-dependent increases in metabolic clearance. These findings support the careful use of dexmedetomidine in this population to minimize adverse events and optimize therapeutic effect during cardiac surgery [22].

Dexmedetomidine produces analgesia through supraspinal and spinal pathways, particularly via α-2-adrenoceptors in the locus coeruleus and dorsal horn of the spinal cord [23]. It reduces hyperalgesia in animal models of neuropathic pain [24]. Sympatholysis occurs through activity at the autonomic ganglia, leading to dose-dependent reductions in blood pressure and heart rate, but is usually mild in nature [25,26,27]. In children, bradycardia of up to 30% from baseline has been observed [14]. At lower doses, dexmedetomidine induces vasodilation, while higher doses can cause vasoconstriction [28,29].

During the perioperative period, dexmedetomidine may reduce stress-related tachycardia and hypertension. While protective against ischemia in some adult studies, its vasoconstrictive effects may also pose a risk [30,31]. Animal studies suggest dexmedetomidine preconditioning reduces infarct size, arrhythmia, and hemodynamic instability [32].

Withdrawal after prolonged infusions in critically ill pediatric cardiac patients has been associated with transient tachycardia, hypertension, and agitation, particularly with abrupt discontinuation. Tachycardia was more frequent in children over one year old (61% vs. 8%, *p* < 0.001) [33]. This may be due to developmental differences in the autonomic nervous system. Older children have a more developed parasympathetic system, so when the sympathetic system is blocked (e.g., by spinal anaesthesia or dexmedetomidine), they experience a marked drop in heart rate and blood pressure. When the block is lifted, their values rise significantly. In contrast, infants, with an immature parasympathetic system, show smaller changes [34].

It has also shown potential for neuroprotection in hypoxic-ischemic injury in developing brains [35]. Renal effects are considered protective, with dexmedetomidine promoting diuresis and preserving cortical perfusion. It may reduce contrast-induced nephropathy and reperfusion injury in animal models [36,37].

## 3. Clinical Applications (Table 1)

### 3.1. Pre-Operative Anxiolysis

As a premedication, dexmedetomidine can be given through the oral, buccal and intranasal route [2]. The efficacy of intranasal atomised dexmedetomidine (at 2.5 µg.kg^−1^) and ketamine as premedication was compared in children undergoing spinal dysraphism surgery. Dexmedetomidine produced significantly better sedation at 20- and 30-min post-administration compared to ketamine, though both drugs showed equivalent results in co-operation during parental separation, intravenous cannulation, and mask acceptance. While heart rate was lower in the dexmedetomidine group, this difference was clinically insignificant [38].

In two randomised control trials consisting of 140 children oral dexmedetomidine and midazolam were compared as premedications in children undergoing elective surgeries. Both drugs provided effective preoperative sedation, with midazolam showing a faster onset. However, dexmedetomidine resulted in significantly lower emergence agitation and higher recovery nurse satisfaction, although it was associated with a slightly higher incidence of hypotension and bradycardia. Overall, dexmedetomidine proved to be a suitable alternative to midazolam [25,26].

### 3.2. General Anaesthesia

Dexmedetomidine has a wide variety of clinical applications. When given intravenously ten minutes before induction of general anaesthesia, it has a dose dependent reduction in required minimum alveolar concentration of Sevoflurane. At doses of 0.6, 0.8, and 1 μg.kg^−1^ IV has been shown to decrease the minimum alveolar concentration (MAC) of sevoflurane by 38%, 48%, and 51%, respectively [39]. A study aimed to evaluate if intraoperative dexmedetomidine (at 1 μg.kg^−1^ IV after induction, followed by an IV infusion at 0.7 μg.kg^−1^.h^−1^) reduces the need for pain relief during and after hypospadias surgery in children. Forty-eight patients were randomly assigned to receive either dexmedetomidine or saline, with both groups receiving standard analgesics. The dexmedetomidine group required significantly less fentanyl, morphine, and paracetamol, and exhibited lower heart rates and blood pressure compared to the placebo group, suggesting an opioid sparing effect [40]. The use of dexmedetomidine during adenotonsillectomies in children with obstructive sleep apnoea resulted in fewer patients requiring rescue fentanyl and morphine. Additionally, dexmedetomidine significantly reduced postoperative opioid requirements and instances of oxygen desaturation without causing adverse hemodynamic effects [41]. Dexmedetomidine reduces non-shivering thermogenesis thus necessitating temperature monitoring and the use of warming devices [2].

### 3.3. Neurosurgery

The efficacy and safety of dexmedetomidine was studied for light sedation in paediatric patients with moyamoya disease (MMD) after revascularization surgery. The results showed that dexmedetomidine provided effective sedation and analgesia, with patients in the dexmedetomidine group being significantly calmer (median Richmond Agitation-Sedation Scale −1.0) compared to the control group (+1.0). Following revascularization surgery in paediatric patients with MMD, a settled patient with no crying is vital [42]. Intraoperative use of dexmedetomidine in children undergoing spinal dysraphism surgery resulted in less intraoperative sevoflurane and fentanyl use, lower postoperative pain and agitation scores, and less postoperative fentanyl with a longer time before the first analgesic dose. Additionally, dexmedetomidine improved recovery without significant haemodynamic instability [43]. It was also successfully used in a case report of two paediatric patients as the sole agent during mapping of the cortical speech area in an awake craniotomy [44].

### 3.4. Airway Procedures

Rigid bronchoscopy for foreign body retrieval in children often requires maintaining spontaneous breathing, achieved with either inhalational agents or total intravenous anaesthesia (TIVA). The addition of intravenous dexmedetomidine can help reduce the required doses of other anaesthetic agents and potentially decrease the risk of coughing, apnoea, and surgical interruptions. A study in China compared a dexmedetomidine/propofol combination (dexmedetomidine bolus of 4 μg.kg^−1^ over 10 min followed by an infusion of 1–2 μg.kg^−1^.h^−1^) and a remifentanil/propofol combination TIVA, both with spontaneous ventilation during airway foreign body removal in children. Both groups had similar induction times, but the remifentanil/propofol group exhibited higher end-tidal CO_2_ and lower respiratory rates. The dexmedetomidine/propofol group demonstrated a more stable respiratory and hemodynamic profile but had a longer recovery time. Remifentanil has the potential to cause chest wall rigidity [45]. This danger is circumvented with the use of the dexmedetomidine/propofol combination. Additionally, the remifentanil/propofol group had a higher incidence of coughing in the post-anaesthesia care unit (PACU), while no significant differences were found in the incidence of adverse events between the groups [46].

The effects of dexmedetomidine and atracurium on intubation conditions were compared in children aged 6–12 years undergoing general anesthesia. The study found no significant difference in intubation quality between the two groups, with all intubations successful. However, dexmedetomidine was associated with lower heart rates and less coughing compared to atracurium, suggesting improved hemodynamic stability. The findings support dexmedetomidine as a safe alternative to atracurium for intubation when the use of neuromuscular blockers is unfavorable [47].

### 3.5. Cardiac Surgery

Dexmedetomidine showed some potential in treating atrial and junctional tachyarrhythmias during the perioperative period of congenital cardiac surgery. Atrial and junctional tachyarrhythmias are common during congenital cardiac surgery, particularly in the postoperative period, and can lead to significant hemodynamic instability. Dexmedetomidine was used as a primary or rescue treatment, achieving heart rate control or conversion to normal sinus rhythm in 93% of cases. While most patients tolerated the drug, adverse effects such as hypotension and brief atrioventricular block occurred in 28% [48].

### 3.6. Use in Neuraxial Anaesthesia

The use of dexmedetomidine as an adjunct in neuraxial anaesthesia had previously been controversial due to varied results with regards to safety and analgesic value. A newer systematic review by Wu et al. of multiple randomised control trials including 1092 patients showed that its use provided significant benefit. Postoperative pain was markedly reduced, and analgesic effect lengthened due to a synergistic effect with local anaesthetics. It was used as an adjunct intrathecally (at 3–15 μg in adults), as an epidural, and as a caudal (at 1–2 μg.kg^−1^). Increased risk of hypotension was not evident, but some risk of bradycardia did exist [49]. Another randomised, double-blind study examined the effects of dexmedetomidine as an adjuvant to bupivacaine in caudal analgesia for paediatric infraumbilical surgeries. Results showed that adding dexmedetomidine significantly prolonged the duration of effective analgesia (9.88 h vs. 4.33 h in the control group) and improved pain scores, without compromising hemodynamic stability [50].

A study of 120 paediatric patients compared the effects of fentanyl, dexmedetomidine (1 μg.kg^−1^), and dexamethasone as adjuvants to local anaesthetics in caudal analgesia for paediatric lower abdominal surgeries. It found that both dexmedetomidine and dexamethasone significantly prolonged postoperative analgesia, reduced pain scores, and lead to fewer patients requiring analgesia compared to fentanyl or local anaesthetics alone. Dexmedetomidine and dexamethasone also resulted in fewer adverse effects than fentanyl, which was associated with higher incidences of vomiting, itching, and respiratory depression [51]. Long-term use of dexamethasone in children has been associated with various adverse effects across multiple organ systems, however, these effects are limited in the context of a single perioperative dose [52,53].

A randomised, blinded study compared the effectiveness of dexmedetomidine versus morphine as adjuvants to bupivacaine in caudal anaesthesia for paediatric thoracic surgeries. Results showed that dexmedetomidine significantly prolonged postoperative analgesia compared to morphine, with lower heart rate, blood pressure, pain scores (Face, Legs, Activity, Cry, and Consolability—FLACC score), and reduced morphine consumption in the dexmedetomidine group. A FLACC score of 4 or more was used to determine the cessation of postoperative analgesic benefit from the caudal. With the addition of morphine postoperative analgesia ranged from 360 to 540 min, with a mean of 414 min. In the dexmedetomidine group the duration of postoperative analgesia ranged from 480 to 840 min, with a mean of 636 min. Adverse effects were similar in both groups, indicating dexmedetomidine as a better option for prolonged pain relief [54].

Another randomised trial examined the effects of adding dexmedetomidine (0.2 μg.kg^−1^) and fentanyl to intrathecal bupivacaine for postoperative analgesia in children undergoing abdominal cancer surgery. The results showed that both adjuvants improved pain relief compared to bupivacaine alone, with dexmedetomidine providing longer analgesia and reducing the need for additional pain medication more effectively than fentanyl. Dexmedetomidine was associated with better overall analgesia [55].

### 3.7. Procedural Sedation

Medical procedures outside of the operating room also regularly result in anxiety and discomfort in the paediatric patient. Patients undergoing imaging studies such as computed tomography (CT), magnetic resonance imaging (MRI) and transthoracic echocardiography frequently require sedation to obtain useful and quality images. A systematic review and meta-analysis assessing the intranasal administration (at 1–3 µg.kg^−1^) of dexmedetomidine as a sole sedative agent resulted in more favourable sedation compared to midazolam. Mild bradycardia and hypotension were associated with dexmedetomidine usage, with none of the patients requiring intervention. A drop in oxygen saturation was reported in only two of the 374 patients that received dexmedetomidine during procedural sedation, which was corrected with a position change or supplemental oxygen [27].

A randomised control trial compared the sedative, hemodynamic, and respiratory effects of dexmedetomidine and midazolam in 80 children undergoing MRI. Dexmedetomidine resulted in better sedation quality, a higher rate of adequate sedation, and less need for rescue medications compared to midazolam. Both drugs caused a decrease in heart rate, mean arterial pressure, and ventilatory frequency, but dexmedetomidine showed shorter sedation onset and fewer side effects [56]. Locally dexmedetomidine does appear in the South African Society of Anaesthesiologists (SASA) paediatric guidelines for the safe use of procedural sedation and analgesia for diagnostic and therapeutic procedures in children. Intravenous (0.5–1 μg.kg^−1^ bolus then 0.2–1 μg.kg^−1^.h^−1^) and intranasal (1–4 μg.kg^−1^) dosing are described [57].

### 3.8. Post-Operative Benefits

A meta-analysis has shown efficacious use of dexmedetomidine in curbing emergence delirium post general anaesthesia in the paediatric population. In groups that received Sevoflurane alone, emergence delirium was reported in 40% of patients. Where Sevoflurane was used with dexmedetomidine, the incidence of emergence delirium was assessed at 12.8% of patients [58]. Another study using an intraoperative infusion of 0.2 μg.kg^−1^.h^−1^ dexmedetomidine decreased the frequency and incidence of emergence delirium in children after general anaesthesia using sevoflurane without lengthening time to extubation or discharge. The dexmedetomidine group had an incidence of emergence delirium in 26% whereas the control group had an incidence of 60.8% [59].

The use of dexmedetomidine during adenotonsillectomies in the paediatric population has shown promising effect. The intra-operative use of an opioid versus dexmedetomidine showed that the pain point system scale score and incidence of apnoea and desaturation post-operatively were significantly less in the latter [41,60]. Several studies have demonstrated the efficacy of dexmedetomidine in preventing postoperative nausea and vomiting (PONV) in children. A meta-analysis of randomised controlled trials revealed that dexmedetomidine significantly reduced the incidence of PONV compared to placebo in paediatric patients undergoing surgeries such as strabismus correction [61].

### 3.9. Sedation/Analgesia in the Intensive Care Unit (ICU)

The value of dexmedetomidine has been demonstrated in sedation of the paediatric population in the intensive care unit. A prospective randomised control trial found that an infusion of 0.5 µg.kg^−1^.h^−1^ dexmedetomidine resulted in more superior sedation than midazolam at 0.22 mg.kg^−1^.h^−1^. This was evidenced by a reduced need for morphine boluses, less morphine required in a 24-h period and less patients with an inadequate Ramsay sedation score of 1 in the dexmedetomidine group [62]. A retrospective study of mechanically ventilated and spontaneously breathing patients that underwent cardiothoracic surgery admitted to the cardiac intensive care unit showed that dexmedetomidine yielded promising results. At infusion rates between 0.2 to 0.75 µg.kg^−1^.h^−1^ effective sedation and analgesia was achieved in 93% and 83% of patients respectively [63].

Effective analgesia and sedation in burn patients can be difficult to achieve due to various factors [64,65]. A retrospective study of paediatric burns patients revealed that where opioid and benzodiazepine-based sedation failed to achieve its goal at safe dosages, dexmedetomidine as an adjunct had bridged the gap whilst avoiding unwanted effects such as withdrawal and respiratory depression [66]. A randomised control trial carried out at three paediatric intensive care units in Italy showed that the prevalence of withdrawal syndrome was somewhat reduced in children receiving dexmedetomidine that underwent at least 5 days of benzodiazepine and opioid infusions. The dexmedetomidine group showed a prevalence of 77.8% compared to 90.9% in the placebo group [67].

Dexmedetomidine offered notable benefits in sedation and analgesia in the neonatal intensive care unit, providing effective pain control—particularly postoperative pain—with minimal respiratory depression and some potential neuroprotective effects. Adverse drug reactions were very limited: only 3% of preterm and 2% of term infants experienced mild bradycardia, and hypotension rates were similarly low. Importantly, higher doses, longer treatment duration, and gestational age (preterm vs. term) were not associated with a higher risk of adverse drug reactions [68].

**Table 1:** Overview of dexmedetomidine use in pediatric patients, including routes, dosages, onset and duration of action across various clinical contexts.

Clinical Application	Route of Administration	Dose	Onset of Action	Duration of Action	References
Premedication	Buccal, intranasal	2–3 µg.kg^−1^ (intranasal)	20–30 min	~1–2 h	[2,38]
Intraoperative use	Intravenous (IV)	0.3–4 µg.kg^−1^ slow bolus, followed by 0.2–2 µg.kg^−1^.h^−1^ infusion	5–10 min (bolus)	Context-sensitive half-time varies with infusion duration	[6,40,46]
Regional use	Epidural, caudal, intrathecal	1–2 µg.kg^−1^ (caudal/epidural); 0.2 µg.kg^−1^ (intrathecal)	~15–20 min	~4–10 h (prolonged analgesia)	[49,55]
Procedural Sedation	Intranasal, IV	1–4 µg.kg^−1^ (intranasal); 0.2–6 µg.kg^−1^.h^−1^ (IV infusion) after 0.5–8 μg.kg^−1^ slow IV bolus	15–20 min (intranasal)	~1–2 h	[1,27,57]
ICU Sedation	Intravenous (IV)	0.2–0.75 µg.kg^−1^.h^−1^ infusion	5–10 min	Context-sensitive half-time varies with infusion duration	[64]
Use in Term and Preterm Neonates	IV, intranasal	0.33 µg.kg^−1^.h^−1^ (IV infusion) after 0.6 µg.kg^−1^ IV loading dose	5–10 min (IV)	Longer due to reduced clearance rates in neonates	[16]

## 4. Conclusions and Future Perspective

Dexmedetomidine has demonstrated significant promise as a versatile sedative and analgesic agent in paediatric anaesthesia and critical care. Its pharmacological profile—marked by α2-adrenergic agonism, minimal respiratory depression, and neuroprotective properties—makes it highly suitable for diverse clinical applications in children, despite its off-label status. While mild bradycardia and hypotension are common, serious adverse events are rare and manageable. Its use in neonates and infants requires careful dosing due to immature metabolic pathways.

Rigorous, large-scale randomized controlled trials are necessary to define optimal dosing, safety thresholds, and long-term outcomes in various paediatric age groups, especially neonates and preterm infants. Dexmedetomidine stands at the forefront of a paradigm shift in paediatric sedation and analgesia, offering a safer, more physiologically harmonious option for children. With further research and formal endorsement, it is poised to become a standard of care across paediatric anaesthesia and critical care domains.
